# Elevated DNA Polymerase Delta 1 Expression Correlates With Tumor Progression and Immunosuppressive Tumor Microenvironment in Hepatocellular Carcinoma

**DOI:** 10.3389/fonc.2021.736363

**Published:** 2021-11-11

**Authors:** Shuai Zhao, Cuicui Wei, Haijia Tang, Han Ding, Bing Han, Shuxian Chen, Xiaoling Song, Qiang Gu, Yichi Zhang, Wangrui Liu, Jian Wang

**Affiliations:** ^1^ Department of Transplantation, Xinhua Hospital Affiliated to Shanghai Jiao Tong University School of Medicine, Shanghai, China; ^2^ Department of Outpatient, Affiliated Hospital of Youjiang Medical University for Nationalities, Baise, China; ^3^ Department of Integrated Medicine, Nanjing University of Chinese Medicine, Nanjing, China; ^4^ Department of Oncology, Xinhua Hospital Affiliated to Shanghai Jiao Tong University School of Medicine, Shanghai, China; ^5^ Department of General Surgery, Xinhua Hospital Affiliated to Shanghai Jiao Tong University School of Medicine, Shanghai, China; ^6^ Affiliated Maternity and Child Health Care Hospital of Nantong University, Nantong, China

**Keywords:** POLD1, hepatocellular carcinoma, prognosis, immune microenvironment, biomarker

## Abstract

**Background and Objective:**

Hepatocellular carcinoma (HCC) is one of the most common cancers worldwide, and the DNA polymerase delta (POLD) family is significantly related to cancer prognosis. This study aimed to explore the significance of the POLD family in HCC *via* the DNA damage repair (DDR) pathway.

**Methods:**

Data mining was conducted using bioinformatics methods. RNA sequencing and clinicopathological data were collected from The Cancer Genome Atlas, GTEx database and the Gumz Renal cohort. Statistical analyses were also performed in cancer samples (n>12,000) and the Affiliated Hospital of Youjiang Medical University for Nationalities (AHYMUN, n=107) cohort.

**Results:**

The POLD family (POLD1–4) was identified as the most important functional component of the DDR pathway. Based on the analysis of independent cohorts, we found significantly elevated POLD expression in HCC compared with normal tissues. Second, we investigated the prognostic implication of elevated POLD1 expression in HCC and pan-cancers, revealing that increased POLD1 levels were correlated to worse prognoses for HCC patients. Additionally, we identified 11 hub proteins interacting closely with POLD proteins in base excision repair, protein-DNA complex and mismatch repair signaling pathways. Moreover, POLD1 mutation functioned as an independent biomarker to predict the benefit of targeted treatment. Importantly, POLD1 expression was associated with immune checkpoint molecules, including CD274, CD80, CD86, CTLA4, PDCD1 and TCGIT, and facilitated an immune-excluded tumor microenvironment. Additionally, we confirmed that elevated POLD1 expression was closely correlated with the aggressive progression and poor prognosis of HCC in the real-world AHYMUN cohort.

**Conclusion:**

We identified a significant association between elevated POLD1 expression and poor patient survival and immune-excluded tumor microenvironment of HCC. Together, these findings indicate that POLD1 provides a valuable biomarker to guide the molecular diagnosis and development of novel targeted therapeutic strategies for HCC patients.

## Introduction

Hepatocellular carcinoma (HCC) is the most common type of primary liver cancer, with over half a million new cases diagnosed worldwide each year. HCC is the most malignant form of liver cancer, which derives from the aggressive proliferation of liver epithelial cells (1). As the sixth leading cause of cancer-related mortality globally, HCC accounts for 4.7% of all cancer deaths. In 2020, over 910,000 people were diagnosed with HCC, and its incidence is continuously rising (2). In China, the incidence of HCC is particularly high, accounting for 55% of the total number of HCC patients worldwide.

Several molecular pathways are implicated in HCC carcinogenesis, including vascular endothelial growth factor receptor (VEGFR), TP53 and Akt/mTOR pathways (3). Among several pathogenic factors, cirrhosis underlies most cases of HCC, and hepatitis C virus (HCV) infection also causes cirrhosis and HCC. However, the eradication of HCV-related cirrhosis does not prevent the development of HCC (4, 5). Thus, more effective HCC treatments are needed.

Historically, serum alpha-fetoprotein (AFP) and diagnostic imaging were the primary diagnostic methods for HCC. However, the poor prognosis of patients owing to the late diagnosis of HCC is unacceptable, and AFP levels are not significantly elevated in most small and early-stage HCCs (6). Recently, several studies have focused on identifying promising biomarkers for early detection (7, 8). Currently, targeted drugs, such as sorafenib, are used as a first-line treatment strategy for the personalized treatment of advanced HCC (9). However, exploring unknown targets and pathways is important to achieve more significant survival benefits for advanced HCC patients who cannot be cured through surgery.

DNA damage repair (DDR) pathways consist of multiple interconnected cellular signaling networks that are activated in response to DNA damage. DDR is correlated with genomic instability, tumor mutational burden in HCC and immune cell function (10). Several studies have shown that the development of cancer is mainly driven by defects in DDR. Therefore, inhibitors targeting kinases involved in DDR, such as AZ20 and M3814, have been investigated for the treatment of cancers in clinical trials (11).

The DNA polymerase delta (POLD) family consisting of POLD1, POLD2, POLD3 and POLD4 is an important mediator of DNA repair during chromosome replication, which is of great significance to the maintenance of DNA structure stability (12). *POLD1* encodes the catalytic subunit of DNA polymerase delta, which is involved in both polymerase and 3’ to 5’ exonuclease activities. POLD1 plays a crucial role in DNA replication and DNA double-strand break repair, together with the accessory proteins POLD2, POLD3 and POLD4 for full activity (13, 14). POLD2 encodes the 50-kDa catalytic subunit of DNA polymerase delta, assisting POLD1 in the process of DDR.

In addition, POLD3 and POLD4 may catalyze the repair of damaged replication forks *via* break-induced replication (15). Recent studies have shown that the loss or reduced expression of POLD3 predicts the occurrence and progression of colorectal cancer and other malignant tumors (16, 17). The decreased expression of POLD3 is associated with the pathogenesis of HCC by enhancing the proliferation and invasion abilities of tumor cells (18).

However, the relationship between the expression of POLD family members and the prognosis of HCC remains poorly understood. Based on the role of the POLD family in the maintenance and repair of DNA, we hypothesized that POLD expression significantly affects the prognosis of HCC *via* the DDR pathway. Our study aims to identify a biomarker with significant potential to improve the diagnosis and prognosis of HCC.

## Methods

### Patients and Tissue Samples From Online Databases and Real-World Cohorts

The large-scale cohorts in this study included RNA sequencing data and accompanying clinicopathological data for 423 HCC patients collected from The Cancer Genome Atlas (TCGA, http://www.cancer.gov) and 10 patients with HCC from the Gumz Renal cohort in the Oncomine (http://www.oncomine.com) database. The threshold was set as follows: *P*-value<0.05, gene rank>10% and data type: mRNA, Cancer *vs*. Normal.

The Affiliated Hospital of Youjiang Medical University for Nationalities (AHYMUN, Guangxi, China) cohort consisted of 107 patients diagnosed with HCC in the Department of Hepatology, Affiliated Hospital of Youjiang Medical University for Nationalities, from June 2009 to August 2018. Clinicopathological data were collected from pathology reports or electronic medical records. Samples of HCC and normal liver tissues were collected during surgery and then processed and stored at the AHYMUN tissue bank before experiments.

### Immunohistochemistry (IHC) Staining Analysis

IHC was performed with an anti-POLD1 antibody-N-terminal (ab226848, Abcam) at a 1:500 dilution. IHC staining was conducted in accordance with the manufacturer’s instructions as previously described (19). Based on the IHC staining intensity and density, two experienced and independent pathologists/clinicians evaluated the overall IHC score (from 0 to 12), with a score of 0 to 3 indicating negative staining and a score of 4 to 12 indicating positive staining for each tissue.

### Establishment of a Protein-Protein Interaction (PPI) Network and Functional Annotations of POLD-Related PPI Networks

In this study, an online web tool for the retrieval of interacting genes (STRING, http://string-db.org; version 10.0) was used to establish a PPI network of differentially expressed genes (DEGs) related to POLD. Then, Spearman’s correlation analysis was performed to describe the correlation between quantitative variables without a normal distribution. The gene ontology (GO) database was used for functional enrichment analyses of biological processes (BP), molecular functions (MF) and cellular components (CC). Kyoto Encyclopedia of Genes and Genomes (KEGG) and Reactome pathway enrichment databases were used to assess large-scale biologic molecular datasets. The publicly available WEB-based GEne SeT AnaLysis Toolkit (WebGestalt; http://www.webgestalt.org/; Version 6.8) was used to explore the potential function of the POLD-related gene panel (20).

### Abundance and Frequency of POLD Mutations in HCC

To further investigate the role of POLD in HCC and pan-cancers, we analyzed the abundance and frequency of POLD mutations in HCC using cBioportal for cancer genomics (http://www.cbioportal.org/). The frequency of typical gene mutations in HCC according to differential POLD1 expression was also analyzed. Significantly elevated genes between POLD1 altered and unaltered groups were screened and identified using the Limma R package.

### Analysis of Immune Infiltration in the Tumor Microenvironment

We grouped the HCC patients (n=107, AHYMUN cohort) into POLD1^high^ and POLD1^low^ groups using the median POLD expression. Tumor Immune Estimation Resource 2.0 (TIMER 2.0, http://timer.cistrome.org/) was used to analyze the correlation between the abundance of tumor-infiltrating immune cells and POLD expression using Spearman’s test. Then, R software was used to evaluate the interactions between immune checkpoint molecules and tumor-infiltrating lymphocytes (TILs) in human cancer samples from TCGA datasets. Spearman’s test was also used to determine the relationship between POLD1 expression and tumor purity using the ESTIMATE algorithm (21).

### Statistics Analysis

To determine the statistical significance of differential POLD expression between tumor and normal tissues, a Student’s t-test was performed. Kaplan–Meier curves with their 95% confidence intervals (95%CIs) and log-rank tests were applied to evaluate the significance of disease-specific survival (DSS) and overall survival (OS) benefits in separate POLD expression groups and all subgroups classified by tumor microenvironment infiltration characteristics using the Kaplan–Meier Plotter (http://kmplot.com/analysis/index). Univariate and multivariate Cox regression analyses were performed to identify the proper terms to build the nomogram. A forest plot was generated with the “forestplot” R package and used to show the *P*-value, hazard ratio (HR) and 95% CI of each variable. A nomogram was developed based on the results of multivariate Cox proportional hazard analysis to predict the X-year overall recurrence and calculate the risk of recurrence for individual patients. Moreover, a Sankey diagram was used to explore the relationship between pathological factors and patient survival.

All statistical analyses were performed using SPSS software (version 23.0, Inc, Chicago, IL), GraphPad Prism 8.0, R software (version 3.4.3), or online web tools. All hypothetical analyses were two-sided, and *P*<0.05 was considered statistically significant.

## Results

### Identification of Critical DDR Pathway Genes and Their Prognostic Implications in HCC

**Figure 1:**
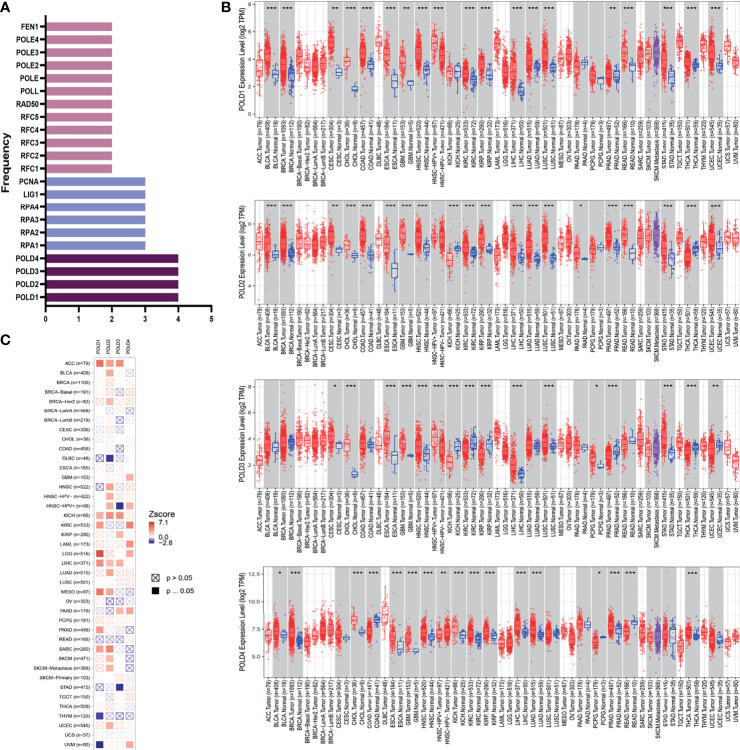
Identification of POLD family members as significant factors in DDR pathways and their prognostic implication in cancer. **(A)** Genes involved in base excision repair, nucleotide excision repair, mismatch repair, homologous recombination, non-homologous end-joining and Fanconi anemia pathways were evaluated, and POLD family members were identified in all four pathways. **(B)** The expression levels of POLDs were significantly higher in HCC tumor tissues compared with normal tissues in more than 12,000 samples. *p < 0.05, **p < 0.01, ***p < 0.001. **(C)** Heatmap analysis indicated that POLD1 has the most significant influence on HCC prognosis compared with POLD2, POLD3 and POLD4.

This study was conducted in three stages (**Figure S1**). To determine the most significant genes in the DDR pathway, we first screened genes involved in base excision repair, nucleotide excision repair, mismatch repair, homologous recombination, non-homologous end-joining and Fanconi anemia pathways. We arranged these genes according to the number of pathways they are involved in and found that the POLD family participated in four pathways, indicating the important value of POLDs in DDR **(** **Figure 1A** **)**. Next, the differential expression in POLD family members between >12,000 tumor and normal tissues was assessed. The expression level of POLDs was significantly higher in HCC tumor tissues compared with normal tissues **(** **Figure 1B** **)**. Furthermore, POLD1 had the highest Z-score in the heatmap compared with POLD2, POLD3 and POLD4, indicating its greater significance for HCC prognosis **(** **Figure 1C** **)**. Based on these results, we mainly explored the important role of POLDs in DDR and the effect of POLD expression on the development and prognosis of HCC.

### Prognostic Role of POLDs in HCC

**Figure 2:**
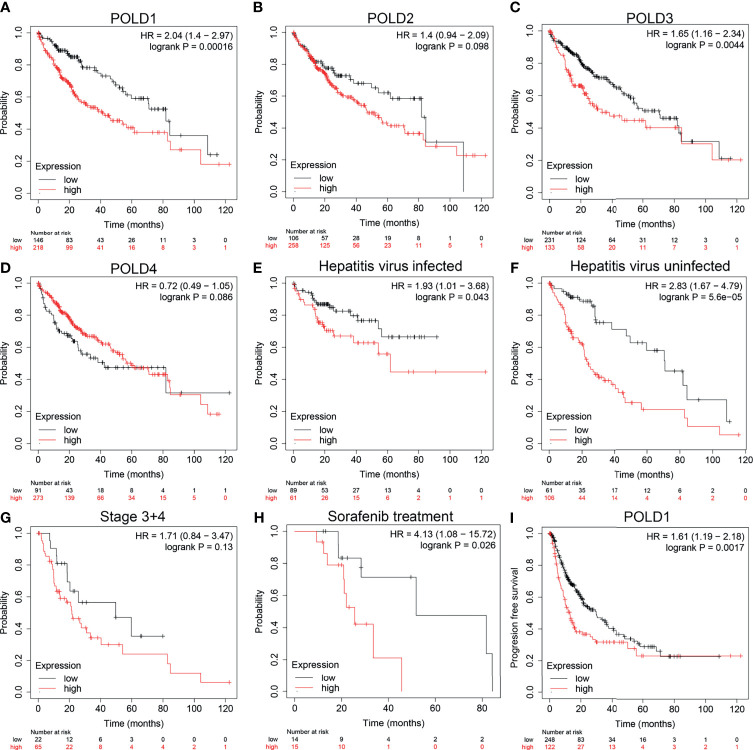
High_ expression of POLD1 is associated with poor survival of HCC patients. **(A–D)** High expression of POLD1 predicted poor OS of HCC patients. The expressions of POLD2, POLD3 and POLD4 had no influence on OS. **(E–H)** Analysis of subgroups (hepatitis virus infection, clinicopathological staging and sorafenib use) in 364 HCC patients from a TCGA cohort indicated that high POLD1 expression was significantly_ linked with poor prognosis. **(I)** High expression of POLD1 was connected with poor PFS in HCC patients (n = 370).

To further confirm the research direction, we performed Kaplan–Meier analysis of 364 patients from the TCGA cohort. High POLD1 expression remarkably predicted a worse OS of HCC patients, whereas patients with higher expression of POLD2, POLD3 and POLD4 did not exhibit significantly shorter OS compared with lower expression patients **(** **Figures 2A–D** **)**. Then, we conducted subgroup survival analyses of POLD1 expression based on hepatitis virus infection, clinicopathological staging and sorafenib use in 364 patients with HCC **(** **Figures 2E–H** **)**. The results suggested that elevated POLD1 expression significantly predicted poor OS in the hepatitis virus infected and uninfected subgroup. However, for HCC patients with an advanced stage, POLD1 expression was not significantly associated with prognosis (HR=1.71, *P*=0.13) but predicted significantly worse outcomes for those receiving sorafenib treatment (HR=4.13, *P*=0.026). Additionally, high POLD1 expression was remarkably related to poor PFS in 370 HCC patients **(** **Figure 2I** **)**.

### POLD1 Significantly Predicts Survival and Aggressive Clinicopathological Parameters for HCC Patients

**Figure 3:**
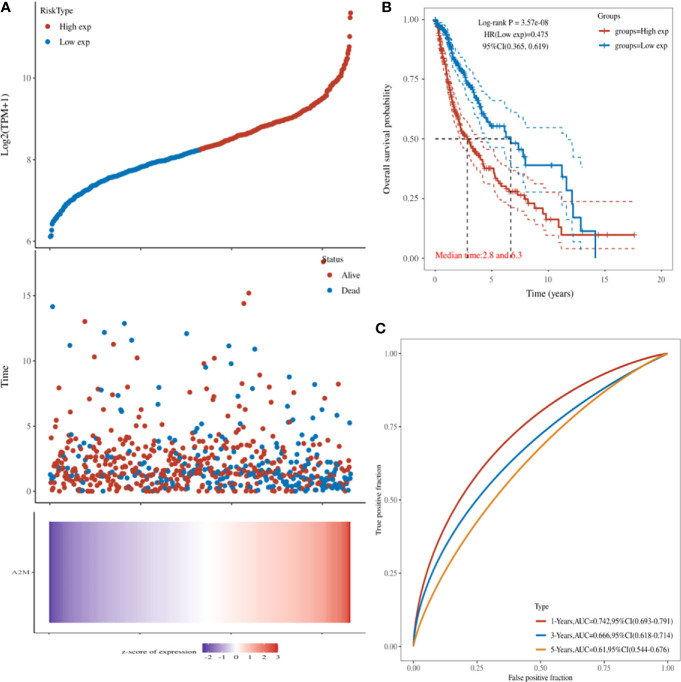
POLD1 predicts survival of HCC patients. **(A)** HCC patients with high POLD1 expression show a significantly higher risk of death compared with those with low POLD1 expression. The z-score confirmed that high expression of POLD1 was associated with a higher mortality. **(B)** Kaplan–Meier curve demonstrated that high POLD1 expression was significantly correlated with worse OS; the median survival was 2.8 years in the POLD^high^ group and 6.3 years in the POLD^low^ group (n = 371 total patients). **(C)** ROC curve demonstrated the high sensitivity and specificity of the diagnostic and prognostic value of POLD1 expression.

Next, we explored the relationship between the expression of POLD1 and the survival of HCC patients. HCC patients with higher POLD1 expression experienced a significantly increased risk of death, and the z-score of POLD1 expression confirmed that elevated POLD1 levels were associated with higher mortality **(** **Figure 3A** **)**. The Kaplan–Meier curve also demonstrated that high POLD1 expression led to worse OS in 371 patients, with a median survival of 2.8 years in the POLD^high^ group and 6.3 years in the POLD^low^ group **(** **Figure 3B** **)**. The high sensitivity and specificity of the independent diagnostic and prognostic value of POLD1 expression were shown by the ROC curve **(** **Figure 3C** **)**. However, the accuracy decreased over time [1-year area under the curve (AUC)=0.742, 3-year AUC=0.666 and 5-year AUC=0.610], suggesting that POLD1 expression more accurately predicted the prognosis of early-stage HCC patients compared with those with advanced stages.

**Figure 4:**
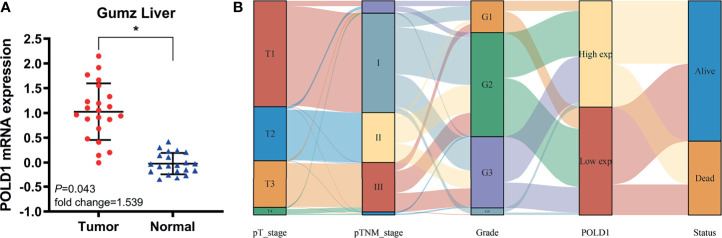
POLD1 predicts aggressive clinic-pathologic parameters in HCC patients. **(A)** Significantly elevated POLD1 expression level was observed in HCC samples compared with normal samples in the Gumz Liver cohort (n = 22; *P* = 0.043). *p < 0.05. **(B)** The Sankey diagram shows the relationship between tumor grades, stages, POLD1 expression level and survival. Low tumor grade and early stage HCC patients tended to have lower expression of POLD1, while high tumor grade and advanced stage were related to the high expression of POLD1.

Next, we assessed the Gumz Liver cohort consisting of differential RNA-seq data from HCC and normal samples. The results revealed a significantly elevated POLD1 expression level in HCC compared with normal samples (n=22; *P*=0.043; **Figure 4A**). The Sankey diagram shows the relationships between tumor grades, stages, POLD1 expression levels and survival. Low-tumor grade and early-stage HCC patients tended to have reduced expression of POLD1, whereas high tumor grades and advanced stages were related to the increased POLD1 expression **(** **Figure 4B** **)**.

### Differential Expression of POLD1 in Pan-Cancers and Its Prognostic Value

Based on HCC tissues and adjacent normal tissues from TCGA (n=423) and GTEx (n=533) datasets, we first compared the POLD1 expression level between pan-cancers and normal tissues. Significant differential expression of POLD1 was commonly observed between cancer tissues and adjacent normal tissues in pan-cancers, such as GMB and STAD, and the expression of POLD1 was significantly higher in the HCC tumor group compared with the normal group **(** **Figures S2A, B** **)**. Second, to explore the prognostic implications of POLD1 expression in pan-cancers, we performed Cox regression analysis **(** **Figures S2C, D** **)**. POLD1 expression was closely correlated with OS in several cancers, such as PRAD (HR=9.25; *P*=2.7e-04) and MESO (HR=2.71; *P*=1.7e-06), and DSS in KIRC (HR=1.93; *P*=7.4e-04) and ACC (HR=3.48; *P*=2.6e-06).

### Construction of a Prediction Model Using Cox Regression and Nomogram Analysis

**Figure 5:**
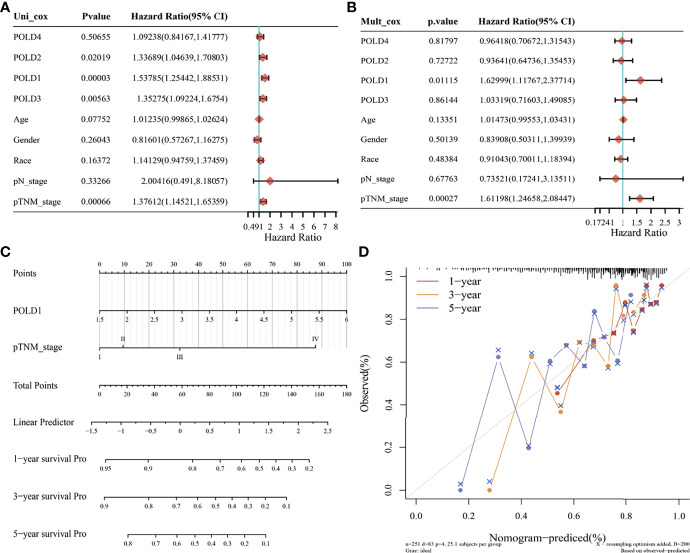
Prediction model based on Cox regression and nomogram analysis. **(A)** Univariate Cox regression analysis indicated that POLD1, POLD2, POLD3 and pTNM stage were closely related to the survival of HCC patients (*P* < 0.05). **(B)** Multivariate Cox regression revealed a significant effect of POLD1 expression (HR = 1.63, *P* = 0.011) and pTNM stage (HR = 1.61, *P* < 0.01) on the prognosis of HCC. **(C)** We used a nomogram to evaluate the prognosis of HCC with a prediction model of POLD1 expression and pTNM stage. **(D)** a graphical representation of the factors was provided by nomogram to calculate the risk of recurrence for an individual patient. The prediction is more accurate in short-term survival prediction (C-index = 0.682, *P* < 0.001).

The univariate Cox regression analysis suggested that POLD1, POLD2, POLD3 and pTNM stages were closely related to the survival of HCC patients (*P*<0.05). Because of the interaction between these factors, multivariate Cox regression revealed a significant effect of POLD1 expression (HR=1.63, *P*=0.011) and pTNM stage (HR=1.61, *P*<0.01) on the prognosis of HCC **(** **Figures 5A, B** **)**. Together, our findings have demonstrated a consistent relationship between high POLD1 expression and advanced clinicopathological staging. Thus, we included POLD1 expression and pTNM stages in a prediction model to evaluate the prognosis of HCC using a nomogram **(** **Figure 5C** **)**. Finally, the nomogram provided a graphical representation of the factors, which was used to calculate the risk of recurrence for an individual patient based on the points associated with each risk factor, and the model was more accurate in short-term survival prediction (C-index=0.682, *P*<0.001; **Figure 5D**).

Univariate Cox regression analysis indicated that POLD1, pTNM staging and grade were closely related to the survival of HCC patients (*P*<0.05), and multivariate Cox regression revealed the significant effects of POLD1 expression (HR=1.54, *P*=0.0005) and pTNM staging (HR=1.27, *P*=0.016) on the prognosis of HCC (**Figures S3A, B**). These findings further confirm the relationship between high POLD1 expression and advanced clinicopathological staging in HCC prognosis. Therefore, we included POLD1 expression and pTNM stage in a nomogram to assess the prognosis of HCC patients (**Figure S3C**). The model is more accurate in short-term survival prediction (**Figure S3D**).

### Landscape of POLD1 Mutation and Related Genes in HCC

**Figure 6:**
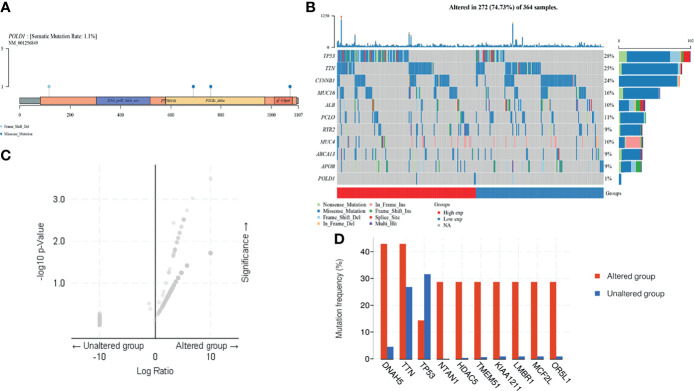
POLD1 mutation analysis in HCC. **(A)** Mutation in POLD1 mainly occurs in POLBc_delta and zf-C4pol. **(B)** Mutations in genes that are commonly mutated in HCC, including *TP53*, *TTN*, *CTNNB1*, *MUC16*, *ALB*, *PCLO*, *RYR2*, *MUC4*, *ABCA13*, *APOB* and *POLD1*, were explored in 272 cases classified based on POLD1 expression. The highest mutation frequency was found in *TP53* (28%) and *TP53* mutation occurred more frequently in the POLD1^high^ group than the POLD1^low^ group. **(C–D)** Significant differentially expressed genes between POLD1 alteration and unaltered groups were identified and included up-regulated genes such as *DNAH5*, *TTN*, *TP53*, *NTAN1*, *HDAC5*, *TMEM51*, *KIAA1211*, *LMBR1*, *MCF2L* and *OR*5*L1* and the down-regulated gene *TP53*.

In addition, to investigate the underlying role of POLD1, we explored the potential value of POLD1 mutation and other related genes based on multi-omics data. First, we found that the mutation was mainly due to POLD1 missense mutation on POLBc_delta and zf-C4pol **(** **Figure 6A** **)**. However, the mutation frequency was only 1.1% in the TCGA cohort. Second, we analyzed the frequency of mutations in common genes in HCC according to differential POLD1 expression in 272 cases, including TP53, TTN, CTNNB1, MUC16, ALB, PCLO, RYR2, MUC4, ABCA13, APOB and POLD1. The highest mutation frequency was found in TP53 (28%). Interestingly, the POLD1^high^ group exhibited significantly more TP53 mutations compared with the POLD1^low^ group, suggesting the importance of the TP53 expression level in clinical treatment and tumor prognosis **(** **Figure 6B** **)**. Then, we explored significant DEGs between the POLD1 altered and unaltered groups and found that DNAH5, TTN, TP53, NTAN1, HDAC5, TMEM51, KIAA1211, LMBR1, MCF2L and OR5L1 were markedly upregulated, whereas TP53 expression was significantly decreased in the altered group compared with the unaltered group **(** **Figures 6C, D** **)**.

### PPI Network Establishment and Functional Enrichment Analysis

**Figure 7:**
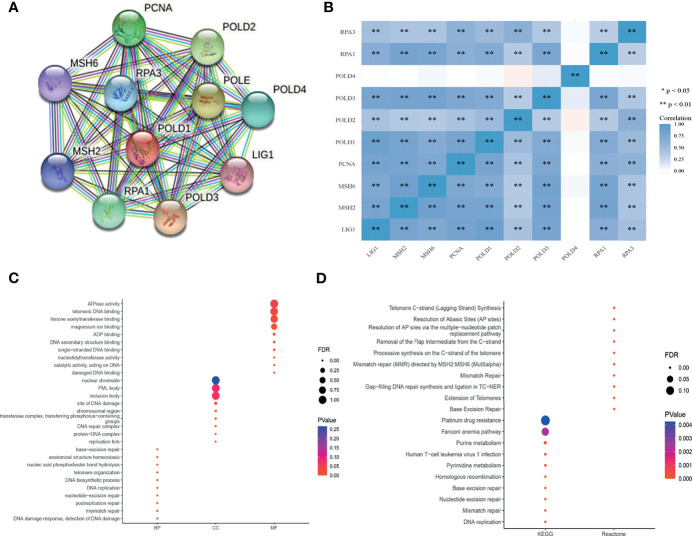
PPI network establishment and functional enrichment analysis. **(A)** A protein–protein interaction network_ was constructed and hub proteins were identified, including POLD1, POLD2, POLD3, POLD4, PCNA, MSH2, MSH6, RPA1, RPA3 and LIG1. **(B)** Except for POLD4, the other ten hub genes showed strong correlations with each other in Pearson’s test. **p < 0.01. **(C–D)** GO function annotation, KEGG pathway and Reactome pathway enrichment analyses were used to analyze the hub genes. The results identified mismatch repair and DNA replication in the biological process, replication fork and protein–DNA complex in cellular components, damaged DNA binding and nucleotidyltransferase activity in molecular function, nucleotide excision repair and base excision repair in KEGG, and extension of telomeres in Reactome analyses.

To explore the mechanism of POLD1 in the DDR pathway, we constructed a PPI network and identified a gene panel of critical genes, including POLD1, POLD2, POLD3, POLD4, PCNA, MSH2, MSH6, RPA1, RPA3 and LIG1 **(** **Figure 7A** **)**. Additionally, we studied the correlation between these genes. Except for POLD4, the other ten hub genes showed a close linear association with each other (Spearman’s test; **Figure 7B** **)**. To analyze the function of these genes, we conducted pathway enrichment analyses, including GO (BP, CC and MF), KEGG and Reactome **(** **Figures 7C, D** **)**. We identified several critical factors, such as mismatch repair and DNA replication in BP, replication fork and a protein–DNA complex in CC, damaged DNA binding and nucleotidyltransferase activity in MF, nucleotide excision repair and base excision repair in KEGG and extension of telomeres in Reactome.

### POLD1 Expression Predicts the Abundance of Immune Cells and Immune Checkpoint Molecules in the HCC Microenvironment

**Figure 8:**
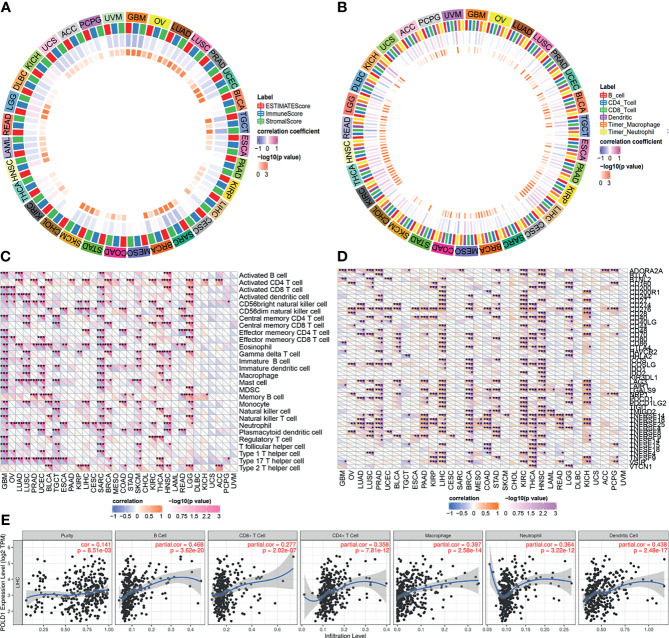
Correlation between POLD1 expression and immune cells. **(A)** High expression of POLD1 was closely associated with tumor purity, immune score and stromal score in pan-cancers. **(B)** Immune cells, including B cells, T cells, dendritic cells, macrophages and neutrophils, were significantly associated with POLD1 expression in many cancers in varying degrees, especially in HCC. **(C–D)** POLD1 was related to the abundance of neutrophils and CD56 natural killer cells in HCC, and POLD1 expression showed strong relationships with most immune checkpoint molecules, including CD274, CD80, CD86, CTLA4, PDCD1 and TCGIT. *p < 0.05, **p < 0.01, ***p < 0.001. **(E)** A scatter plot shows the close relationship between POLD1 expression level and tumor purity (HR=0.141), B cells (HR=0.468), CD8^+^ T cells (HR=0.277), CD4^+^ T cells (HR=0.358), macrophages (HR=0.397), neutrophils (HR=0.364) and dendritic cells (HR=0.438).

High expression levels of POLD1 were found to be closely associated with tumor purity, immune scores and stromal scores in pan-cancers **(** **Figure 8A** **)**. In addition, several immune cells, including B cells, T cells, dendritic cells, macrophages and neutrophils, were significantly associated with POLD1 expression to varying degrees in different cancers, especially HCC (r^2^>0.4, **Figure 8B**). Moreover, POLD1 expression was related to the abundance of neutrophils and CD56 natural killer cells in HCC **(** **Figure 8C** **)**. Meanwhile, POLD1 levels exhibited a strong relationship with most immune checkpoint molecules, including CD274, CD80, CD86, CTLA4, PDCD1 and TCGIT **(** **Figure 8D** **)**. The scatter plot shown in **Figure 8** demonstrates the close relationship between POLD1 expression levels and tumor purity (HR=0.141), B cells (HR=0.468), CD8^+^ T cells (HR=0.277), CD4^+^ T cells (HR=0.358), macrophages (HR=0.397), neutrophils (HR=0.364) and dendritic cells (HR=0.438). Overall, these findings suggested that POLD1 expression was correlated with the infiltration of several immune cells and reshaped the immune-excluded microenvironment.

### Validation of Differential POLD1 Expression and Its Prognostic Value in the AHYMUN Cohort

**Figure 9:**
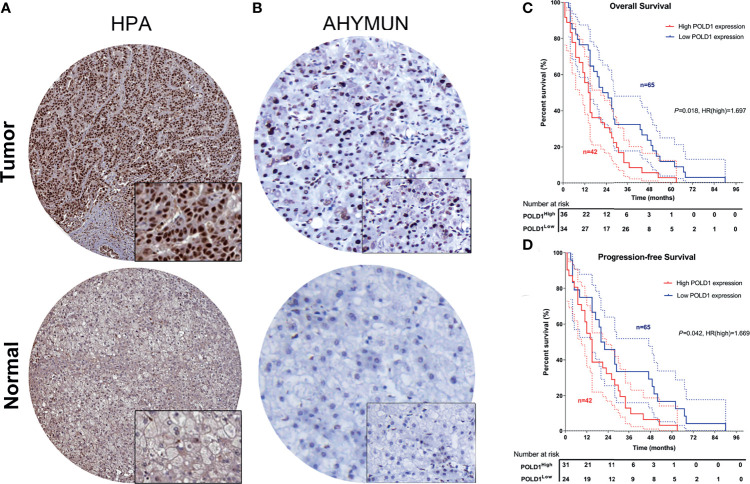
Validation of differential POLD1 expression and its prognostic value in the AHYMUN cohort. **(A, B)** IHC analysis of samples from the AHYMUN cohort showed that the expression of the POLD1 protein was significantly higher in HCC than in normal liver tissues. **(C, D)** Prognostic implications of POLD1 expression in 107 HCC patients from the AHYMUN cohort indicated that a high POLD1 protein level was significantly associated with worse OS (*P* = 0.018, HR = 1.697) and PFS (*P* = 0.042, HR = 1.669).

To validate the increased expression of POLD1 in HCC samples compared with normal liver tissues, we first collected samples from HPA and explored the prognostic implications of POLD1 expression in 107 HCC patients from the AHYMUN cohort. The nuclear expression of POLD1 was significantly higher in HCC than adjacent normal tissues based on the HPA database and AHYMUN cohorts **(** **Figures 9A, B** **)**. Additionally, the results suggested that increased protein expression of POLD1 was closely associated with worse OS **(** *P*=0.018, HR=1.697) and PFS (*P*=0.042, HR=1.669; **Figures 9C, D** **)**.

## Discussion

HCC is the most common primary liver cancer and has a poor prognosis. Over the past few decades, the incidences of liver cancer and liver cancer-related deaths have increased (4, 22). However, the treatment options for advanced liver cancer are very limited, and strategies for advanced personalized treatment of HCC are lacking (23). Therefore, better understanding of the mechanisms underlying HCC is critical because these findings may help identify novel treatments for HCC patients. In this study, we examined the potential prognostic value of POLD in HCC and evaluated its role in the tumor immune microenvironment.

POLD1 interacts with POLD2, POLD3, and POLD4 to form the POLδ holoenzyme together with replication factor C and proliferating cell nuclear antigen. POLD2–4 are smaller subunits, and POLD1 plays a major role in the biochemical activity of the polymerase (24). Consistent with these data, we found that POLD1 showed a more significant influence on tumor prognosis than the other three POLD proteins and therefore we focused on POLD1 for the subsequent analysis.

POLδ has important proofreading capabilities conferred by exonuclease activity and is also involved in repairing DNA damage, including nucleotide excision repair, double-strand break repair, base excision repair, and mismatch repair (25, 26). Multiple studies have linked germline and sporadic mutations in POLD1 and other subunits of POLδ with human pathologies (27, 28). For example, mutations in POLδ in mice and humans lead to genome instability, mutant phenotypes, and tumorigenesis. In addition, mutations in the proofreading domain of POLD1 have been identified as the root cause of some hereditary cancers and these mutations may affect treatment management. Recent studies have shown that loss-of-function alterations in DDR genes are associated with human tumorigenesis (29). Germline mutations in POLE and POLD1 have been shown to predispose patients to multiple colorectal polyps and a wide range of neoplasms (30). A previous study showed that at least 1 in 92 primary liver cancer patients had DDR gene mutation (31). The landscape of DDR mutations and their association with genetic and clinicopathologic features suggest that PLC patients with altered DDR genes may be rational candidates for precision treatment (32). Therefore, in addition to the increased expression level of POLD1, POLD1 and POLE mutations may function as independent biomarkers to predict the benefit of targeted treatment.

The expression levels of POLD family members in tumor and normal tissues were explored. According to previous research, the increased expression of POLD3 indicated a poor prognosis of HCC patients, although not as significantly as POLD1 (33). POLD3 plays a specialized role in the repair of damaged replication forks, indicating that POLD3 activity may be particularly relevant for cancer cells enduring high levels of DNA replication stress (12). The cellular depletion of POLD1 or POLD3 resulted in differential genome instability manifested by DNA double-stranded breaks (17, 34).

Human cancers can be divided into three types according to the anti-tumor immune response status, or the immune phenotype: inflamed, immune-excluded, and immune desert status. Inflamed cancers generally refer to tumors with high PD-L1 expression in cancer cells and more immune cells and tumor infiltrating lymphocytes in the tumor; these tumors are sensitive to immune checkpoint inhibitors. Immune-excluded tumors are tumors in which the stroma shows a large number of T cells, but the T cells cannot penetrate the stroma and infiltrate the tumor because of the strong inhibitory microenvironment. These tumors often do not respond well to immune checkpoint inhibitors. Immune desert tumors lack the infiltration of T cells and immune cells even in the interstitium, which is a described as an “immune desert”. In our analysis, we found that POLD1 is closely related to the expression of B cells, CD8+ T cells, CD4+ T cells and macrophages. These results suggest that POLD1 may play an important role in the immune-excluded tumor microenvironment. We speculate that this may be because POLD1 is involved in DNA cleavage repair, which affects the tumor microenvironment.

There are some advantages of this study. First, this research contained independent HCC cohorts, including the TCGA database (n=423), GTEx database and Gumz Renal cohort (n=10), and the real-world AHYMUN cohort (n=107). Second, we demonstrated the value of significantly elevated POLD expression for HCC prognosis and identified POLD1 as the most valuable gene for further analysis. Third, at both the mRNA and protein levels, we validated the association between POLD1 expression and HCC prognosis. Moreover, ~12,000 tumor samples from TCGA database were collected, and the effect of POLD1 expression on prognosis was verified in pan-cancers. Finally, functional enrichment analysis was performed. The role of POLD1 in the infiltration of immune cells in the tumor microenvironment was demonstrated, which may guide cancer treatment and targeted drug development.

This study has several limitations. First, there is a high degree of heterogeneity between the patient groups in this study. Therefore, in the next study, we will select patients from multiple regions for multi-center research. Second, this was a retrospective study. Finally, we will further study the demographic, clinical and pathological details of the population.

Our study has demonstrated a link between elevated POLD1 expression and patient survival and the tumor microenvironment in HCC. In the next study, we will explore the role and mechanism of POLD in HCC progression through cytological tests and animal experiments. We will also analyze the potential mechanisms of POLD, POLδ, and DNA cleavage repair in HCC through high-throughput sequencing and other methods. These findings will help provide new insights into the pathogenesis of HCC and new ideas for the personalized treatment of HCC patients.

## Conclusion

This study first investigated the molecular and clinical role of the POLD family and revealed the significant relationship between elevated POLD1 expression and the poor survival and immune-excluded tumor microenvironment of HCC patients. Together, these findings support the use of POLD1 as a biomarker to guide the molecular diagnosis and development of novel targeted therapeutic strategies for HCC patients.

## Data Availability Statement

The original contributions presented in the study are included in the article/**Supplementary Material**. Further inquiries can be directed to the corresponding authors.

## Ethics Statement

The study design and experimental procedures were performed in accordance with the Declaration of Helsinki. The study was approved by the ethics committee of the Affiliated Hospital of Youjiang Medical College for Nationalities (Baise, Guangxi Province, China). Written informed consent was obtained from all participants.

## Author Contributions

WL and SZ carried out the molecular genetic studies, participated in the sequence alignment and drafted the manuscript. JW and SC carried out the immunoassays. HD, CW and BH participated in the sequence alignment. XS and QG participated in the design of the study and performed the statistical analyses. HT, SZ, YZ, WL, and JW conceived the study, participated in the study design and coordination and helped to draft the manuscript. All authors contributed to the article and approved the submitted version.

## Funding

This study was supported by grants from the Medical Engineering Cross Fund of Shanghai Jiaotong University (No. YG2021QN50), the National Natural Science Foundation of China (No. 82103520), and the 2020 Nantong Municipal Science and Technology Plan (No. JCZ20138).

## Conflict of Interest

The authors declare that the research was conducted in the absence of any commercial or financial relationships that could be construed as a potential conflict of interest.

## Publisher’s Note

All claims expressed in this article are solely those of the authors and do not necessarily represent those of their affiliated organizations, or those of the publisher, the editors and the reviewers. Any product that may be evaluated in this article, or claim that may be made by its manufacturer, is not guaranteed or endorsed by the publisher.

## Supplementary Material

Supplementary Figure 1 Flowchart of the study.

Supplementary Figure 2 High expression of POLD1 in pan-cancers compared with normal liver tissues. **(A, B)** POLD1 expression levels between pan-cancers and normal tissues were compared in the TCGA (n = 423) and GTEx (n = 533) datasets. POLD1 expression was differentially expressed in cancer tissue and adjacent normal tissue in pan-cancer, and the expression of POLD1 was significantly higher in the HCC tumor group compared with the normal group. **(C, D)** The POLD expression level in pan-cancers and normal tissue based on data from the TCGA and GTEx databases. The results are unremarkable in some cancers. **(C, D)** Cox regression analysis showed that POLD1 expression is closely correlated with OS in many cancers.

Supplementary Figure 3 Prediction model based on Cox regression and nomogram analysis of POLD1. **(A)** Univariate Cox regression analysis suggested that POLD1, age, grade, race and pTNM stage were closely related to the survival of HCC patients (*P *< 0.05). **(B)** Multivariate Cox regression revealed the significant effect of POLD1 expression and pTNM stage on the prognosis of HCC. **(C)** We used a nomogram to evaluate the prognosis of HCC with a prediction model of POLD1 expression and pTNM stage. **(D)** A graphical representation of the factors was provided by nomogram to calculate the risk of recurrence for an individual patient. The prediction is more accurate in short-term survival prediction.
