# Supplementary figures and images for: Elevated DNA Polymerase Delta 1 Expression Correlates With Tumor Progression and Immunosuppressive Tumor Microenvironment in Hepatocellular Carcinoma
Source: Front Oncol. 2021 Nov 11;11:736363. doi: 10.3389/fonc.2021.736363 (PMC8632622; doi:10.3389/fonc.2021.736363)

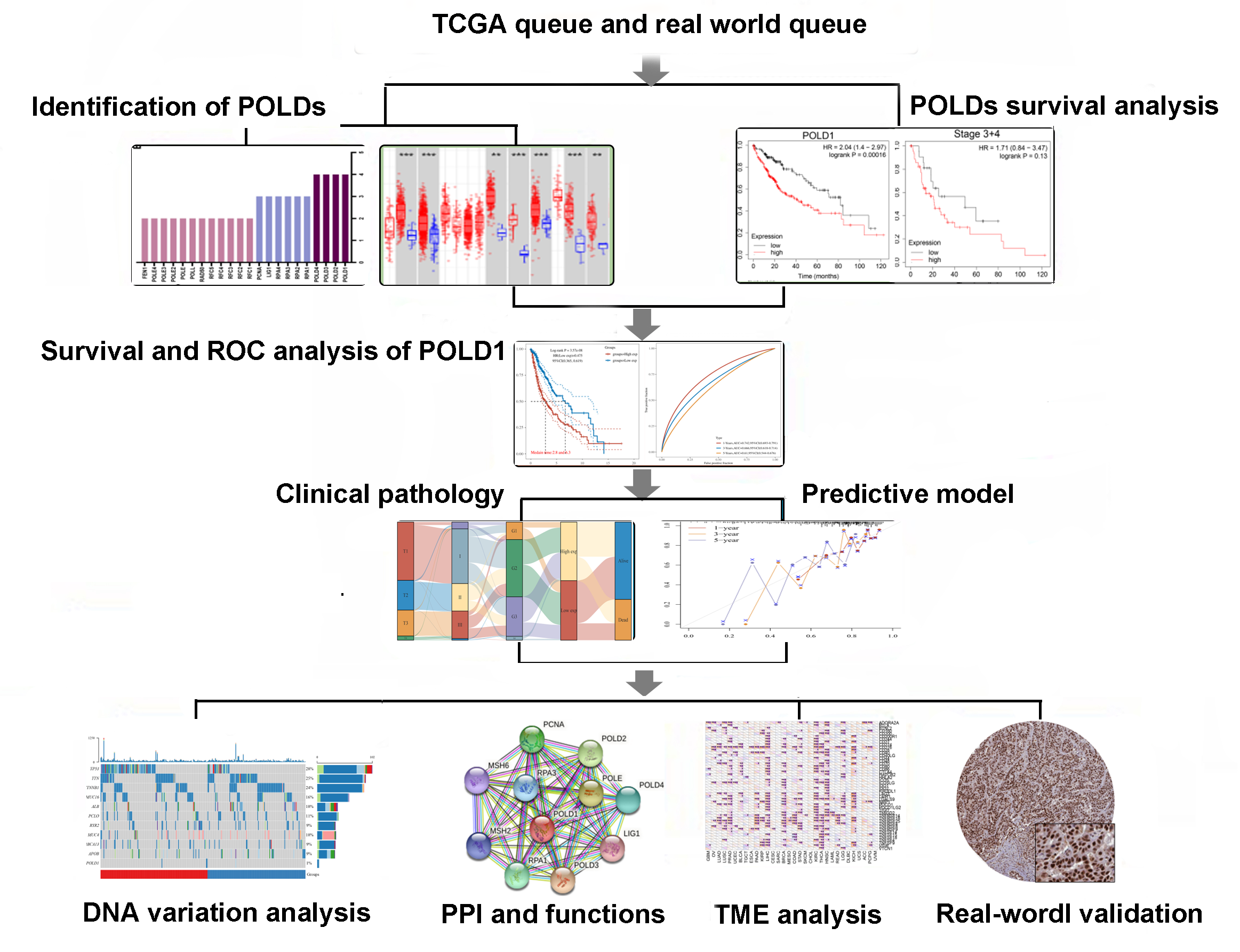

Supplement: Supplementary Figure 1 — Flowchart of the study. [file Image_1.tif]

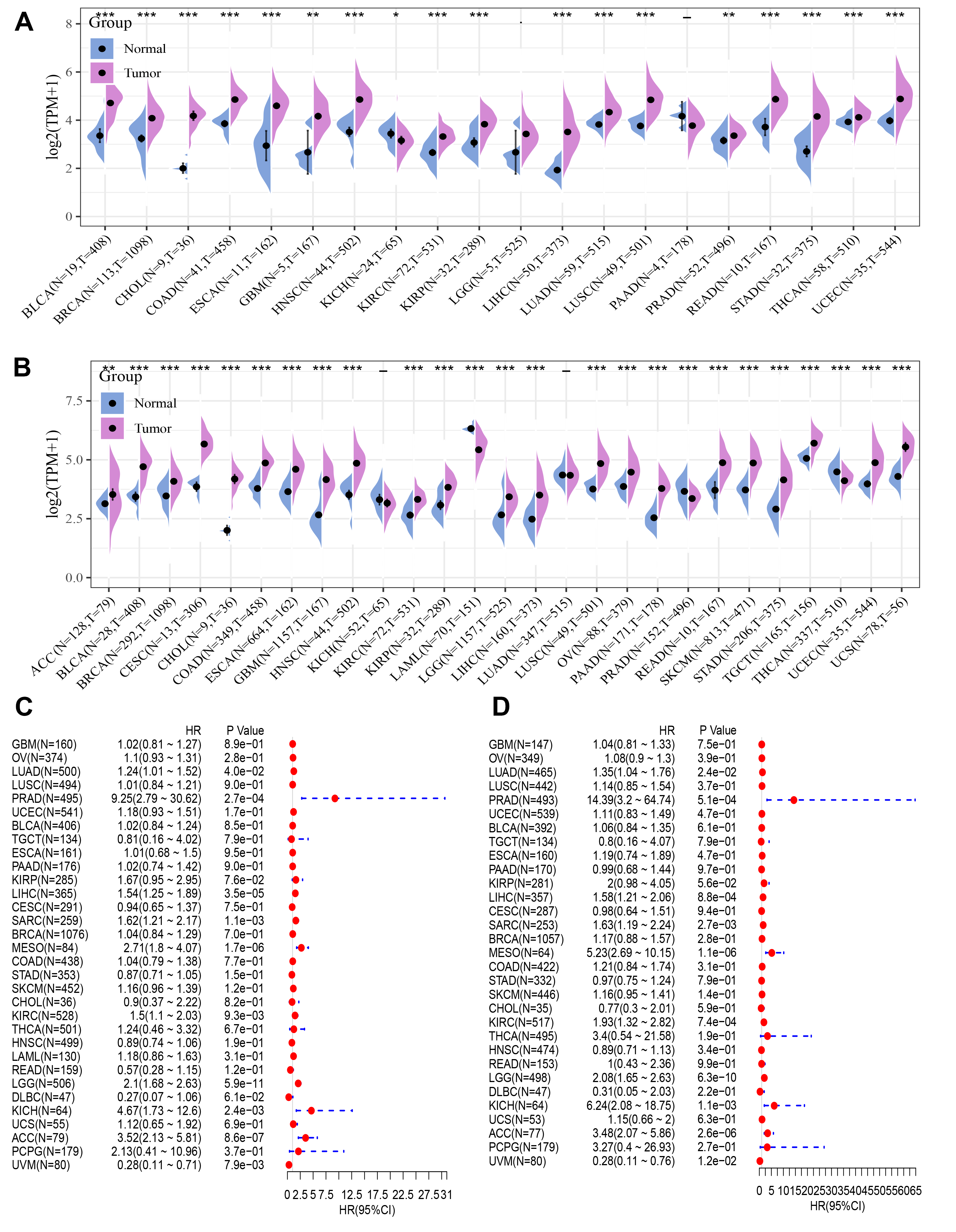

Supplement: Supplementary Figure 2 — High expression of POLD1 in pan-cancers compared with normal liver tissues. (A, B) POLD1 expression levels between pan-cancers and normal tissues were compared in the TCGA (n = 423) and GTEx (n = 533) datasets. POLD1 expression was differentially expressed in cancer tissue and adjacent normal tissue in pan-cancer, and the expression of POLD1 was significantly higher in the HCC tumor group compared with the normal group. (C, D) The POLD expression level in pan-cancers and normal tissue based on data from the TCGA and GTEx databases. The results are unremarkable in some cancers. (C, D) Cox regression analysis showed that POLD1 expression is closely correlated with OS in many cancers. [file Image_2.tif]

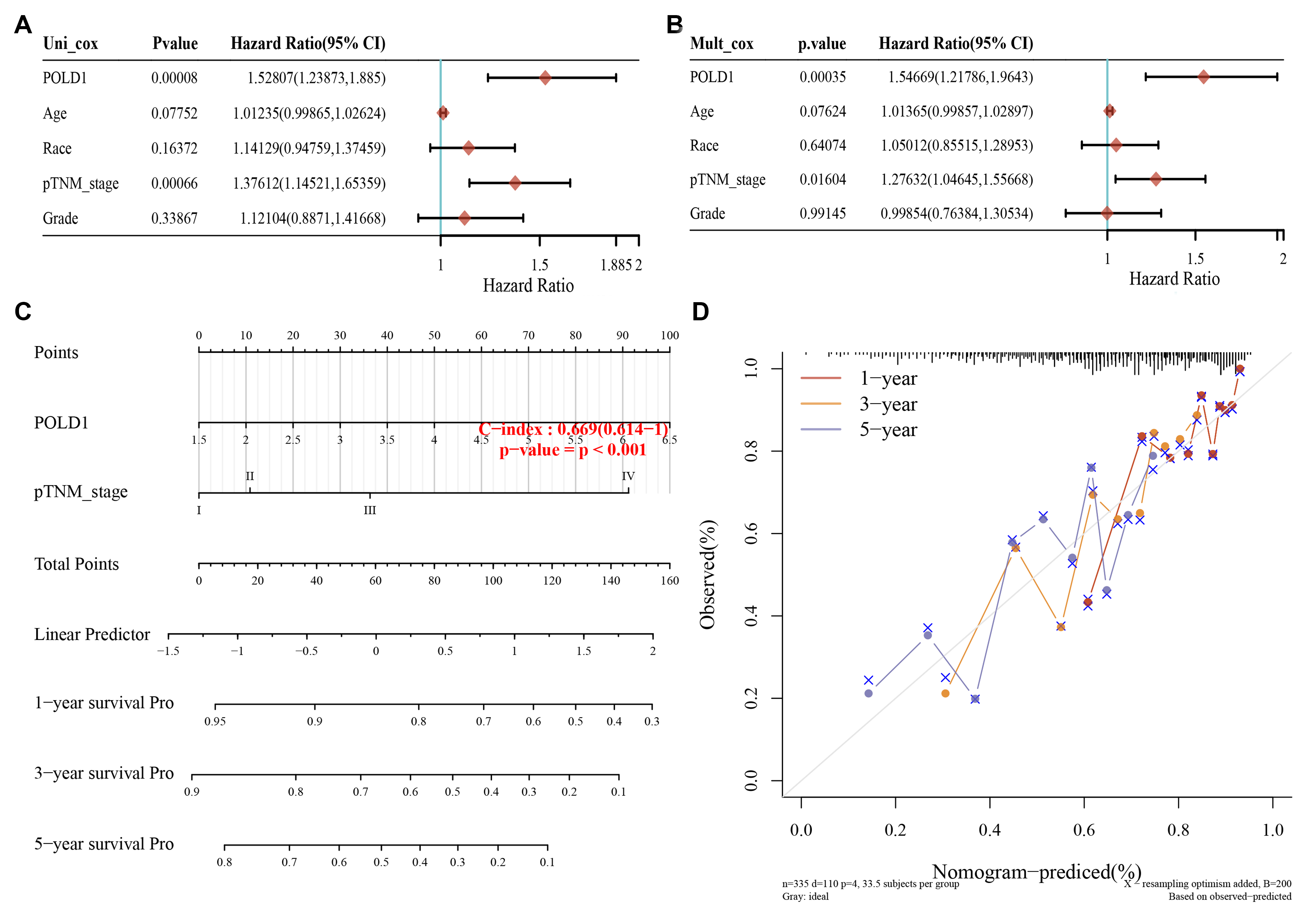

Supplement: Supplementary Figure 3 — Prediction model based on Cox regression and nomogram analysis of POLD1. (A) Univariate Cox regression analysis suggested that POLD1, age, grade, race and pTNM stage were closely related to the survival of HCC patients (P < 0.05). (B) Multivariate Cox regression revealed the significant effect of POLD1 expression and pTNM stage on the prognosis of HCC. (C) We used a nomogram to evaluate the prognosis of HCC with a prediction model of POLD1 expression and pTNM stage. (D) A graphical representation of the factors was provided by nomogram to calculate the risk of recurrence for an individual patient. The prediction is more accurate in short-term survival prediction. [file Image_3.tif]
